# Troubled Waters: The Future of Global Fisheries

**DOI:** 10.1371/journal.pbio.0020113

**Published:** 2004-04-13

**Authors:** Virginia Gewin

## Abstract

Scientists debating how to assess global fisheries are now including studies of long-term ecosystem effects and options for recovery efforts. But is it possible to both conserve and farm the sea?

It is becoming increasingly apparent that the vast blue expanse of ocean—the last frontier—is not as inexhaustible as it once seemed. While we have yet to fully explore the reaches of the sea, technology has granted humans the ability to harvest its wealth. We can now fish anywhere, at any depth, for any species. Like the American frontier range's bison and wolf populations brought to the brink of extinction swordfish and sharks are the ocean's most pursued prizes. The disadvantages associated with the depth and dimensions of this open range, however, have long obscured the real consequences of fishing. Indeed, scientists have the formidable challenge of assessing the status of species whose home covers over 75% of the earth.

Three recent highly publicized papers—a trifecta detailing troubled waters—call attention to overfishing's contributions to the dramatic declines in global fisheries. Delving into the past, Jeremy Jackson and colleagues (2001) combined local historic records with current estimates to detail the ecological impacts of overfishing, Reg Watson and Daniel Pauly (2001) drew attention to distortions of global catches, and Ransom Myers and Boris Worm (2003) highlighted the depletion of the majority of the largest ocean predators. While some have valid criticisms of the assumptions and aggregation of historic data used to assess the global situation, few disagree with the overriding conclusion that humans have drastically altered not only fish biodiversity, but, increasingly, the ocean itself.

Recent reports by the United Nation's Food and Agriculture Organization (FAO) which maintains the world's most complete global fisheries database, appear to validate the conclusions of these studies. The most recent FAO report states that 28% of global stocks are significantly depleted or overexploited, and 47% are either fully exploited or meet the target maximum sustainable yield. Only 24% of global stocks are either under- or moderately exploited. As the sea is increasingly harvested, many ecologists wonder how the ecosystem will continue to function (Jackson et al. 2001). Although economic and social considerations often supercede scientific assessments, science will continuously be called upon to deliver management options that will straddle the needs for conservation and production, even in areas where there is only subsistence fishing (Box 1). As scientists debate the details of global fisheries assessment, they are also including studies of the long-term ecosystem effects and options for recovery efforts. Like was done on the open range, shall we conserve or farm the sea—or both?

## Catches, Collapses, and Controversies

The FAO began keeping fisheries records in 1950. Unfortunately, an enormous amount of data comes directly from each country's fishing industry, which is often biased as a result of unreported discarding, illegal fishing, and the misreporting of harvests. For example, mid-level Chinese government officials seeking promotions systematically enhanced China's fisheries numbers in recent years—which inflated and skewed international catch rates.

The FAO data show that catches, excluding a recent surge in anchoveta and China's suspect numbers, reached a peak of 80 million metric tons in the late 1980s and have since begun to decline. Regional studies validate these trends. “Most of the line fish around the coast of South Africa are depleted to 5%–15% of pristine levels,” says George Branch, a marine biologist from the University of Cape Town (Cape Town, South Africa). Meryl Williams, Director General of WorldFish in Penang, Malaysia, notes that the Asia-specific database called TrawlBase (www.worldfishcenter.org/trawl/) confirms that the region's commercial species have been depleted to 10%–30% of what they were 30–40 years ago.

Obtaining accurate information on highly migratory species is challenging, to say the least. It is not hard to imagine that data quality is the biggest disadvantage to any scientific assessment. Of the 50 managed stocks in the northeast Atlantic Ocean—including invertebrates, sport fishes, and major commercial finfish—data are kept on only one-fifth of the species. There are 250 fish species in the region, but only 55 species are of commercial interest and merit inquiry. “We know next to nothing about noncommercially fished species,” notes Jeff Hutchings, a conservation biologist at Dalhousie University (Halifax, Nova Scotia, Canada). And that is where fisheries have adequate access to current monitoring programs. “With the recent expansion of the Taiwanese and Chinese fleets, we don't have the kind of sampling programs needed for those kinds of fisheries,” says Rick Deriso, a fisheries scientist with the Inter-American Tropical Tuna Commission (IATTC) (La Jolla, California, United States).

Couple these inadequacies with previously unknown bycatch rates (i.e., the fish caught in addition to the target catch) and illegal catches, and it is easy to see that the task is formidable. The FAO estimates that roughly one-quarter of the marine commercial catch destined for human consumption—some 18–40 million metric tons of fish—is thrown back in the sea, a harvested catch that is never utilized or counted. It is estimated that the illegal, unreported, and unregulated (IUU) fisheries surpass allowed fishing quotas by 300%. IUU fishers operate in areas where fishing is not permitted, use banned technologies or outlawed net types, or underreport catches. “The IUU fishery for Patagonian toothfish expanded rapidly in the mid-1990s, likely on the order of 20–30 vessels,” says Andrew Constable, an ecological modeler at the Australian Antarctic Division (Kingston, Australia), who also works with the Scientific Committee of the Commission for the Conservation of Antarctic Marine Living Resources (Hobart, Australia). “These rates of IUU fishing could reduce stocks to threshold levels in some areas in two to five years,” he adds.

Often overlooked is the inescapable fact that even sustainable harvest rates reduce fish populations quickly. “If the goal is a productive fishery, we're automatically talking about up to a 70% decline in population across the board,” says Deriso. The FAO's Chief of Marine Resource Services, Jorge Csirke, states that “from a stock point of view, there is no way to preserve integrity of wild stocks and exploit them at the same time.” Indeed, the United States' National Marine Fisheries Service (NMFS) considers optimal harvest rates to be between 40%–60% of virgin levels. But once fish populations dip below the 10%–20% mark, declines are of serious concern.

**Figure 1:**
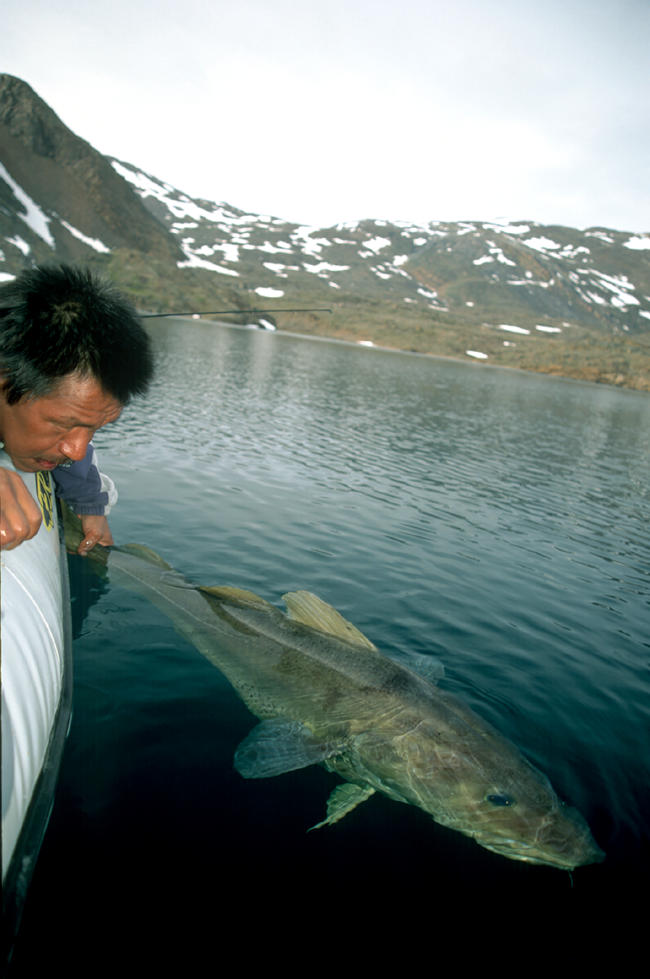
Cod in a High Arctic Lake in Canada These cod resemble those of past Atlantic catches. Measuring 47–53 inches (120–135 cm) long and weighing between 44 and 57 pounds (20 and 26 kg), it is easy to see that today's 16–20 inches (40–50 cm) commercially caught cod are less than half this size. (Photo, with permission, by David Hardie, Dalhousie University.)

Atlantic cod in Canadian waters suffered a total population collapse and are now on Canada's endangered species list (Figure 1). From 2 billion breeding individuals in the 1960s, Atlantic cod populations have declined by almost 90%, according to Hutchings. While advisors called attention to declining cod stocks, Constable notes that by the time a significant declining trend has been detected by traditional catch assessments, stocks are likely to be in poor shape, if not already depleted.

Given the task of compiling data on only the economically important species, fisheries biologists developed a single-species management approach in the 1960s, which assumed that fisheries affect each species in isolation. This approach, although now rife with problems, served the community and the politicians well during the decades of abundant resources. “They brought the approach of single-species management to near-perfection,” says Boris Worm, a marine ecologist at the Institute for Marine Science in Kiel, Germany. A growing discontent with the model, in addition to greater awareness of ecological interactions, however, prompted Worm and his Dalhousie University colleague Ransom Myers to question the sustainability of the single-species approach. Attempting a comprehensive assessment, their widely cited recent paper (Myers and Worm 2003) indicated that the global ocean has lost more than 90% of large predatory fishes, such as marlin, sharks, and rays.

**Figure 2:**
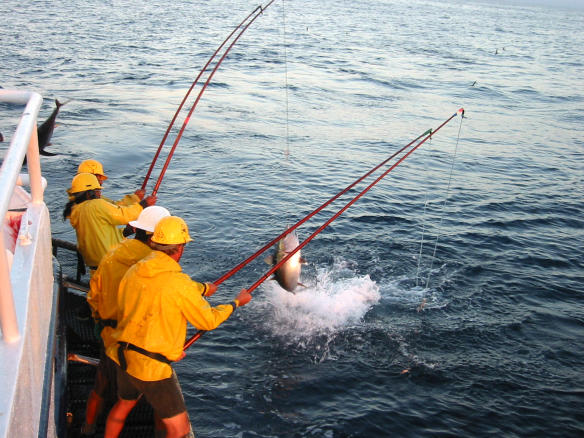
Pole Fishing for Medium-Sized (40–50 lb or 18.1–22.7 kg) Big-Eye Tuna aboard the Live-Bait, Pole-and-Line Vessel *Her Grace* (Photo, with permission, by Kurt Schaefer and Dan Fuller, IATTC.)

However, this new approach to assess fish stocks is not without its critics. Fisheries biologists point out that the nuances of management contained in fisheries data—such as altered fisher behavior, the variable “catchability” of individual species, and altered gear use—were discounted in the Myers and Worm (2003) assessment and led to misinterpretations for some species, notably tropical tunas (Figure 2). A number of tuna biologists have expressed concern that these omissions have left the mistaken impression that all tuna species are among the list of declining predators (Hampton et al 2003). Worm acknowledges that his approach can be improved, but says, “The whole point of our paper was to aggregate species to communities to see what the overall ecosystem is doing.”

## Ecosystem Sustainability

Despite the controversy, most agree that the large predators, particularly sharks, skates, rays, and marlin, are in the most dire straits. Unlike other lower-trophic order species, the wholesale removal of top predators has enormous effects on the rest of the ecosystem. One consequence is that overall reproduction rates can potentially suffer. Fish size, gender, and age at maturity have a substantial impact on individual species' reproduction rates. Since larger fish are the most susceptible to fishing, the population's age structure can shift as individuals, particularly females, are fished out. For example, a 23-inch (59-cm) female vermilion rockfish can produce 17 times the young of a 14-inch (36-cm) fish. Given uncertainties with population dynamics, the fact that basic biological data are missing makes the job even harder. While knowledge of these components is still quite spotty, tuna inventories, for example, have started collecting gender data on catches.

Daniel Pauly, a fisheries biologist at the University of Vancouver (Vancouver, British Columbia, Canada), has shown that increased fishing has caused the industry to “fish down the food web,” or systematically move to lower trophic levels over time as higher ones were depleted (Pauly et al. 1998). The impact to ecosystems is only beginning to be uncovered. “If you fish out an abundant predator, the species that it was eating or competing with will increase,” says Worm. “The problem is that the ecosystem may change in such a way that recovery is inhibited because a species niche space is taken or altered.”

Fisheries science has taken steps to increase the quality of data in recent years. “Traditional fishery models assumed that a fishery was a homogenous thing—like bacteria in a bottle—rather than a spatially diverse system,” says Pierre Kleiber, a fisheries biologist with the Pacific Islands Fisheries Science Center of the NMFS (Honolulu, Hawaii, United States). He adds that recent work accounts for spatial diversity. In addition, fisheries are now dealing with the inherent uncertainty of their work and are factoring that into models and decision-making. “Uncertainty didn't used to be dealt with at all in formulating fishery management advice,” confirms Keith Sainsbury, a marine ecologist with the Commonwealth Scientific and Industrial Research Organisation (CSIRO) (Clayton, South Victoria, Australia), adding that its absence gave rise to an awful lot of troubles. “Traditional models tended to assume perfect data with no holes in it,” says Kleiber. “Now we've tried to craft a model to fit the realities of missing data.”

**Figure 3:**
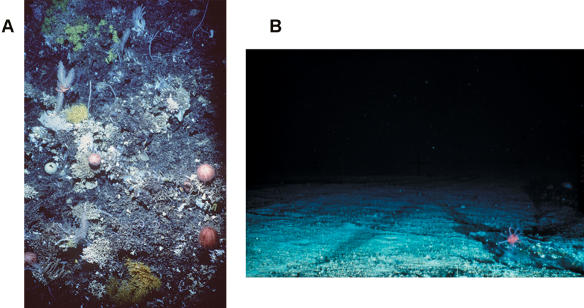
The Effect of Trawling the Seafloor for Groundfish (A) The coral community and seabed on an untrawled seamount. (B) The exposed bedrock of a trawled seamount. Both are 1,000–2,000 meters (1094–2188 yards) below the surface. (Photo, with permission, by CSIRO Marine Research.)

As well as incorporating spatial diversity and uncertainty, researchers are beginning to comprehend the ecological damage caused by different types of fishing gear. Indeed, trawling the bottom of the seafloor for groundfish can destroy a half-acre footprint of habitat (Figure 3). Detailed reports document that, depending on the habitat's stability, bottom trawling can not only remove fish from seafloor habitats, but alter bottom relief such that it compromises the ability of other fish to survive (NRC, 1002). In Australia, for example, lingcod rely on undisturbed bottom relief to lay their eggs, while other groundfish species depend on complex seafloor habitats for the majority of their food.

“Science is getting more realistic, but it is getting more difficult,” says Branch. Ecological models are far more complex than traditional fisheries models, says Csirke, adding that more model variables make it more difficult to apply to fisheries, an industry whose focus is, understandably, not conservation. Despite its incorporation into national fisheries policies, ecosystem-based management remains a loosely defined term. It is not a well-defined concept because it is not possible to optimize every species, says Deriso.

An additional concern to scientists is that of biomass resilience in the face of environmental changes. Francisco Chavez, a biologist with the Monterey Bay Aquarium Research Institute (Moss Landing, California, United States), recently demonstrated that over a 25-year period, warmer and cooler Pacific waters tilt the distribution of anchoveta versus sardines, both open-ocean dwellers (Chavez et al. 2003). Indeed, El Niño influenced the crash of the heavily fished Peruvian anchoveta industry in the late 1970s. These examples illustrate how susceptible fisheries are to environmental fluctuations. When the biomass of a population is reduced, it is much more sensitive to environmental change. We do not know how environmental fluctuations like these will affect the natural production of young fish, says Kleiber, expressing the concern that without a better understanding of climate, fisheries scientists end up trying to estimate moving targets.

In the end, many scientists have their doubts about the influence of science on decision-making. “My personal view is that it's naïve to think that modifying and improving models will necessarily lead to improved natural resource management,” says Simon Jennings, a fisheries biologist with the United Kingdom's Centre for Environment, Fisheries and Aquaculture Science in Lowestoft. Indeed, the International Council for the Exploration of the Seas (Copenhagen, Denmark) recently recommended a total ban on North Sea and Irish Sea cod stocks, based on single-species assessment. Although the more intensive ecosystem-based models could not have produced a more stringent recommendation, politicians allowed harvests at roughly half of last year's catch.

## To Conserve or to Farm?

While lowering fisheries' effort seems the most logical approach to the recovery of depleted fisheries, social and economic concerns often stymie political action. Yet demand for seafood continues. Therefore, scientists also are investigating both conservation and alternative production options.

Given the social, economic, and political problems associated with that, managers have often used closures to help a hard-hit species recover. In many cases, however, the recovery time for exploited species is longer than once thought (Hutchings 2000). “Based on the available information, it is not unusual for fish populations to show no or little recovery even after 15 years,” says Hutchings. “All else being equal, we predict the earlier the age of maturity, the faster the rate of recovery,” he adds. And that depends on environmental conditions as well. “In the case of Antarctic species, some overexploited populations remain at less than 5% pre-exploitation abundance after 30 years,” says Constable.

One management strategy to recover species is to create marine protected areas (MPAs), zones that restrict all removal of marine life (Box 2). A number of marine ecologists are staunch supporters of MPAs for both conservation and fishery's recovery. What looked like sustainability in the past were fisheries out of our reach—naturally protected areas—says Pauly, adding that our increasing ability to harvest fisheries necessitates the creation of MPAs now. In theory, these areas are refugia for fishes to reproduce, spilling over not only healthy adults but also potentially transporting thousands of viable young—seeding surrounding waters. To date, less than 1% of the ocean's area is protected, which hinders the ability to conclusively determine if spillover rates have the predicted impact on fishery's recovery.

A review of 89 studies of MPAs by Ben Halpern, a student at the University of California, Santa Barbara (Santa Barbara, California, United States), demonstrated that the average number of fish inside a reserve increases between 60%– and 150% (Halpern 2003). In addition, 59% of the sites had increased diversity. While the numbers inside the reserves look good, the crucial condition of larval spillover has yet to be proven. Most scientists involved in the debate agree that MPAs should be one component in an overall management scheme, but worry that until the crucial element of fishing effort is resolved, MPAs may just displace the vast industrial fleets.

In terms of simply producing fish for global food needs, aquaculture (also known as fish farming) is another, increasingly popular, option. In 2001, the European Union produced 17% of total fishery's production via aquaculture. These numbers are projected to steadily increase, but some question whether aquaculture would be sufficient to supply what has been lost by overexploited fisheries.

Concentrated in coastal areas, aquaculture has aroused numerous concerns. Indeed, in developed countries, most operations grow carnivorous fish, which necessitates growing fish to feed fish. While the process has become more efficient in recent years, due in part to a growing reliance on vegetarian diets, it still takes about 3 pounds (1.36 kg) of fish to create 2.2 pounds (1 kg) of desirable meat (Aldhous 2004). Yet, the total catch of food fish continues to grow, as do concerns about nutrient runoff and estuary pollution resulting from aquaculture. Increasingly, coastal residents often complain about the aesthetics of such activities, and there is also new research that indicates that farm-raised fish harbor more cancer-causing pollutants than wild species (Hites et al. 2004).

To alleviate many of these concerns, open-ocean aquaculture is now being considered. Indeed, the NMFS is set to propose a Code of Conduct for Offshore Aquaculture, which would open up the 200-mile (322-km) United States Exclusive Economic Zone to net pens seaward of coastal state boundaries and authorities. The Sea Grant program in conjunction with interested business, is also currently assessing the carrying capacity of open-water pens as well as their potential environmental impact. Given increased industrial interest and unchanging demand for seafood, many think farming the sea may be around the corner.

Undoubtedly, scientific effort will continue to inform both conservationists and industry about fisheries' capacity and potential recovery options. As attitudes towards fisheries continue to change, increased understanding of the ecological underpinnings should help strike a more informed balance between fisheries' conservation and production. “The big mistake is suggesting that you can manage fish stocks,” says Niels Daan, a biologist with the Netherlands Institute for Fisheries Research (IJmuiden, The Netherlands). “In my opinion, we can only manage human activity.”

##

**Box 1. Fisheries Management in Developing Countries**

While industrial-scale fishing is a growing concern to fisheries biologists, the management of subsistence fishing in developing countries is equally complex. Indonesia alone has 1.3 million fishers. Given the lack of alternative economic options for subsistence fishers, it is much more difficult to reduce fishing because it meets immediate food and resource needs. Local scientists, often lacking in resources, have a much more difficult time assessing the effects and offering advice to governmental fisheries regulators, who have limited political influence. Kenyan researcher Tim McClanahan notes that a main problem is a lack of coordination and respect between traditional and national programs of management. Therefore, he focuses on the fishing gear used. By reconciling the impact of certain fishing gear with traditional knowledge, McClanahan has developed a basis for suggested restrictions deemed acceptable to the local community.

**Box 2. The Establishment of High Profile MPAs**

While MPAs are heavily touted as one of the best management tools to address both conservation and fisheries management, few have been enacted. In 2001, following a strong mandate by the Australian Minister to the Environment and overwhelming political will, the Great Barrier Reef Marine Park Authority (GBRMPA) in Australia established a network of marine protected, or no-take, areas as an ecosystem-based management approach.

In setting up the reserve networks, scientists determined the most effective areas to protect biodiversity with little impact to productivity. “We tried to avoid peak use areas, while protecting at least one-third of each bioregion and minimizing the impact to users of the Great Barrier Reef Park,” says Phil Cadwallader, Director of Fisheries at the GBRMPA.

Off the coast of California, the Channel Islands network of marine reserves, established in April 2003, consists of 13 areas designed to protect biodiversity and critical habitat for breeding fish and to maintain biodiversity. The area has suffered serious declines of red snapper, angel sharks, and abalone, once plentiful off the California coast, over the past decade. Scientists designed the network to protect those productive habitats that would help ensure that larval dispersal was maintained between the individual reserves. Totaling 132 nautical square miles (342 nautical square kilometers), 11 of the areas are no-take reserves—allowing no fishing or harvest of any kind.

## Abbreviations

CSIRO: Commonwealth Scientific and Industrial Research Organisation
FAO: Food and Agriculture Organization
GBRMPA: Great Barrier Reef Marine Park Authority
IATTC: Inter-American Tropical Tuna Commission
IUU: illegal
MPA: marine protected area
NMFS: National Marine Fisheries Service
